# The Gap Between Self-Rated Health Information Literacy and Internet Health Information-Seeking Ability for Patients With Chronic Diseases in Rural Communities: Cross-sectional Study

**DOI:** 10.2196/26308

**Published:** 2022-01-31

**Authors:** Zhuoxin Wang, Yanyan Fan, Hekai Lv, Shanshan Deng, Hui Xie, Li Zhang, Aijing Luo, Fuzhi Wang

**Affiliations:** 1 School of Nursing Bengbu Medical College Bengbu China; 2 School of Health Management Bengbu Medical College Bengbu China; 3 Innovation Team of Health Information Management and Application Research (BYKC201913) Bengbu Medical College Bengbu China; 4 Key Laboratory of Medical Information Research (Central South University) College of Hunan Province Changsha China; 5 The Second Xiangya Hospital Central South University Changsha China

**Keywords:** online, health information, barriers to acquisition, middle-aged patients with chronic diseases, rural community, chronic conditions, chronic, rural, literacy, information seeking

## Abstract

**Background:**

The internet has become one of the most important channels for residents to seek health information, particularly in remote rural areas in China.

**Objective:**

In this study, we aimed to explore the gap between self-rated health information literacy and internet health information seeking ability for patients with chronic diseases in rural communities and to preliminarily evaluate their barriers when seeking health information via the internet.

**Methods:**

Residents from rural communities near Bengbu City and with chronic diseases were included in this study. A self-rated questionnaire was used to evaluate their health information literacy, 3 behavioral competency tasks were designed to preliminarily evaluate their ability to seek health information on the internet and semistructured interviews were used to investigate their barriers to obtaining health information via the internet. A small audiorecorder was used to record the interview content, and screen-recording software was used to record the participants’ behavior during the web-based operational tasks.

**Results:**

A total of 70 respondents completed the self-rated health information literacy questionnaire and the behavioral competence test, and 56 respondents participated in the semistructured interviews. Self-rated health information literacy (score out of 70: mean 46.21, SD 4.90) of the 70 respondents were moderate. Although 91% (64/70) of the respondents could find health websites, and 93% (65/70) of the respondents could find information on treatment that they thought was the best, 35% (23/65) of respondents did not know how to save the results they had found. The operational tasks indicated that most articles selected by the respondents came from websites with encyclopedic knowledge or answers from people based on their own experiences rather than authoritative health information websites. After combining the results of the semistructured interviews with the DISCERN scale test results, we found that most interviewees had difficulty obtaining high-quality health information via the internet.

**Conclusions:**

Although the health information literacy level of patients with rural chronic disease was moderate, they lack the ability to access high-quality health information via the internet. The vast majority of respondents recognized the importance of accessing health information but were not very proactive in accessing such information.

## Introduction

Chronic diseases have become major challenges to global health [1]. According to 2018 data from the World Health Organization, chronic diseases cause 41 million deaths every year, accounting for 71% of all deaths worldwide, and of these, 15 million occur among people aged 30 to 69 years [2]. China has documented significant decreases in the age of patients with chronic disease, and the number of such patients is increasing over time [3]. Previous studies [4] have found that health professionals are the main source of health knowledge for patients with chronic diseases. With the rapid development of information technology in recent decades, an increasing number of patients with chronic diseases choose to obtain health information through the internet [5,6], particularly in the vast rural areas of China [7]. Relevant studies [8] have shown that health-related interventions implemented via the internet can improve the health status of patients with chronic physical diseases. However, numerous studies have shown that the quality of internet health information is not optimal in China [9] or in other countries around the world [10,11]. Additionally, the low level of information literacy among the public in rural areas [12] causes some users to have difficulty in effectively using health information from the internet to improve their health statuses.

In the past decade, some studies [13,14] have suggested that health information literacy should be a key part of public health promotion in China. Health information literacy is the set of abilities that users have to recognize the need for health information, identify possible sources of information, and use those sources to retrieve relevant information; evaluate the quality of information and its applicability to a given situation; and analyze, understand, and use this information to make sound health decisions [15]. Despite the increasing amount of health information available on the internet, patients with limited health knowledge may lack the necessary skills to access the internet or make use of this information. A study [6] examining the relationship between eHealth literacy and health information–seeking behaviors and participation in mobile health research among African Americans found that most participants scored high on eHealth literacy but lacked the ability to distinguish between high- and low-quality health resources on the internet and to use internet information to make health decisions. The study also reported that people with lower education levels were less likely to use the internet to obtain health-related information [6]. Another cross-sectional study [16] found that patients with chronic diseases rely on health care professionals for health information regardless of their level of health literacy and that patients with low levels of health information literacy lacked the ability to use the internet to look up health information (ie, they had low health information–seeking behaviors on the internet). Therefore, in the context of the rapid development of global information technology, improving the public's ability to access quality health information through the internet remains an important element of health information literacy promotion.

The term *internet health information–seeking behavior* refers to the process in which users search for health knowledge or information on the internet to meet their own health information needs and to reduce the uncertainty of their health statuses [17]. A review [18] of existing studies on patients’ internet health information–seeking and its impact on doctor-patient relationships and found that such information seeking could improve doctor–patient relationships. Another study [19] evaluated the characteristics of different types of internet users in seeking web-based health information and found that there were differences in the use of and access to health information among people of different ages, races, and socioeconomic status. Another study [20] found that a large proportion of older adult patients with chronic diseases use the internet to seek health information; the onset time and type of chronic diseases may play an important role in their internet health information–seeking behavior. At the same time, Latino individuals are less likely than white individuals to search for health information but are more likely to use health information to treat disease whereas African American individuals are more likely to use health information to maintain their health [21]. A couple studies in China [22,23] have been conducted on the internet health information–seeking behavior of patients with chronic diseases. A cross-sectional study [22] of 313 hospitalized patients with chronic diseases in Shanghai found that the patients’ attitudes toward health information–seeking were in the middle to upper levels and the patients had a high demand for health information. Another study [23] investigated the health information–seeking behavior of hypertensive patients in Guizhou Province, and the results indicated that the health information needs of hypertensive patients were diverse. The main health information access channels were traditional interpersonal relationship channels (medical personnel), while new media network technology was seldom used; the digital divide is the main cause for this problem. We found that the current research methods for patients with chronic disease in China internet health information–seeking behavior are limited to questionnaire surveys and subjective evaluations. There is a lack of research employing interviews and operational evaluations of the internet health information–seeking behavior of patients with chronic diseases. The lack of such research suggested that we should conduct in-depth evaluations on the barriers faced by patients with chronic diseases in seeking health information on the internet from an objective and extensive research perspective.

In this study, questionnaires, semistructured interviews, and behavioral competency tests were used to explore the gap between self-rated health information literacy and internet health information–seeking abilities for patients with chronic diseases and to preliminarily evaluate their barriers to seeking health information via the internet. These results are expected to provide support for the government and health education institutions to perform internet health information behavioral intervention for patients with chronic diseases in the future.

## Methods

### Research Tasks

#### Overview

This research investigation included 3 tasks. First, a self-rated health information literacy questionnaire was used to evaluate the health information literacy levels of the respondents. Second, 3 operational tasks were designed and administered to test the respondents’ abilities to seek internet health information on a computer or mobile device. Finally, interviews on internet health information–seeking (containing 9 questions) were performed. Before the survey began, 3 experts (doctoral degrees and ample research experience) were invited to evaluate the validity of the survey materials used in this study, and appropriate modifications were made based on the experts’ opinions (Multimedia Appendix 1).

#### Task 1: Self-Rated Health Information Literacy Questionnaire

A self-rated questionnaire was used to assess the health information literacy of respondents. The questionnaire was synthesized and revised from the 10-item Everyday Health Information Literacy scale [24] and modified in 2018 [25]. The scale contains 14 items divided into 4 dimensions: health information consciousness, health information–seeking, health information evaluation, and health information application. A higher score indicates a higher health information literacy level. The questionnaire has been widely used for assessing the health information literacy of Chinese digital immigrants in rural communities [12] and Chinese residents [25] and has shown good reliability, validity, and adaptability for the Chinese population.

#### Task 2: Internet Health Information-Seeking Behavior Ability Tests

Without receiving guidance from the researchers administering the test, the respondents were asked to log on to the internet to search for health information on their own, and respondents’ behavior throughout the entire test was recorded with screen-recording software. In this task, the respondents were asked to complete 3 operational tasks: (1) use the internet to find what they thought was the best health website containing knowledge about chronic diseases; (2) according to their own health conditions, choose and save 2 to 3 articles related to health knowledge they considered valuable; and (3) choose an article that they thought discussed the best treatment for a certain chronic disease and save it (if the respondents had difficulty saving the article, the investigator helped them save it for subsequent analyses).

The DISCERN scale was used to evaluate the quality of the chronic disease treatment articles selected internet by the respondents. The DISCERN scale, developed by the British Library and the University of Oxford [26], is divided into 2 parts. The first part consists of 8 items and has a total score of 40; this part was used to evaluate the reliability of the website. The second part includes 7 items and has a total score of 35; this part was used to test the quality of the articles.

#### Task 3: Semistructured Interviews

The semistructured interviews focused on 2 topics: the retrieval process and strategy and the evaluation of the quality of health information on the internet. The investigator conducted an in-depth exchange with the respondents to understand their criteria for selecting the health information websites, their methods for identifying the quality of information and the difficulties they encountered in obtaining internet health information. The interviews were recorded throughout.

### Participants

The participants of this study were patients with chronic diseases in Xiaobengbu Town, Bengbu City, Anhui Province. Two-stage sampling was used. In the first stage, 10 rural communities in the town of Xiaobengbu were identified using simple random sampling; in the second stage, a general practitioner from the community health service station was invited to select 10 patients with chronic diseases from the health files of community residents by simple random sampling and establish contact with patients by telephone, introduced the research content to them, and invited them to the community health service station to participate in this survey after obtaining their consent.

The participants included in the study met the following criteria: (1) had been living continuously in the rural community for more than 6 months, (2) were between 30 and 65 years old, (3) had been diagnosed with 1 or more chronic diseases, and (4) had experience using the internet. The exclusion criteria were as follows: (1) individuals with physical and mental conditions that made them not suitable for participation in this survey and (2) individuals who did not complete any 1 of the first 2 tasks.

### Preparation for the Investigation

The surveys were conducted in person. Web-based survey software (Tongtai Questionnaire Survey Platform, Beijing Tongtai Technology Development Co Ltd) was used to complete the questionnaire survey, a small audiorecorder (model R2, JingZheng,) was used to record the interview content, and screen-recording software (Windows: KK Lu Xiang Ji, version 2.8; Android: Lu Ping Da Shi, version 3.3) was used to record the participants’ behavior during the internet operational tasks. Before the start of the investigation, the screen-recording software was installed on the computer and tablet computer. After each participant completed the investigation, the investigator exported the recording file and cleared all browsing records and network settings. The duration of the investigation for each participant was limited to be within 1 hour. After each survey, we offered the participant a bucket of cooking oil worth 50 RMB (approximately US $7.74).

### Statistical Analysis

Data entry and preprocessing were performed using Excel (version 2010; Microsoft Inc), and statistical analysis was performed using SPSS software (version 16.0, SPSS Inc). Descriptive statistical analyses were performed by calculating the mean and standard deviation, and intragroup differences were analyzed using the *t* test and Kruskal-Wallis test. *P* values <.05 were considered significant.

### Ethical Approval

This study was approved by the Ethics Committee of the Bengbu Medical College (2017054). The survey was completed anonymously. All potential respondents were contacted personally and thoroughly informed about the aim of the study, data processing, and the use of the data. Participation was voluntary, and participants could refuse to participate.

## Results

### Self-Rated Health Information Literacy

In total, 70 respondents (Table 1) completed task 1 and task 2, among whom 56 participants completed task 3, and 14 participants did not complete the semistructured interview (quit the interview due to loss of interest: n=7; did not give clear answers to the questions: n=5; provided irrelevant answers: n=2).

The average health information literacy score of the respondents was 46.21 (SD 4.89), out of a total possible score of 70; the highest score was 56, and the lowest score was 34. Of the 70 respondents, 91% (n=64) scored at least 40. The subdimension *health information evaluation* had the highest score (mean 14.30, SD 2.98), followed by *health information–seeking* (mean 12.80, SD 2.23), *health information consciousness* (mean 11.20, SD 1.04), and *health information application* (mean 7.91, SD 0.37).

The results of the univariate analysis showed that, except for gender (*P*=.04), education level (*P*=.02), and experience with internet use (*P*=.03), the self-rated health information literacy scores did not differ significantly based on the other sociodemographic factors (age: *P*=.70; occupation: *P*=.23; mobile phone use: *P*=.99; chronic diseases: *P*=.98; time postdiagnosis: *P*=.62).

**Table 1:** Sociodemographic characteristics and self-rated health information literacy scores.

Characteristic			Participants (n=70), n (%)		Self-rated health information literacy score		Chi-square or *t* test^a^ (*df*)		*P* value
**Gender**							–2.135^a^ (68)		.04
	Male	39 (56)		45.13 (5.08)					
	Female	31 (44)		47.58 (4.35)					
**Age**							0.156 (1)		.70
	31 to 45 years old	27 (39)		46.44 (4.27)					
	46 to 60 years old	33 (47)		46.33 (5.20)					
	61 to 65 years old	10 (14)		45.20 (5.79)					
**Education**							7.799 (2)		.02
	Primary school or below	9 (13)		42.78 (5.21)					
	Middle school or technical school	56 (80)		47.09 (4.56)					
	University	5 (7)		42.60 (4.39)					
	Postgraduate and above	0 (0)		0.00 (0.00)					
**Occupation**							4.264 (3)		.23
	Employees of enterprises and public institutions (including separation and retirement)	29 (41)		47.24 (4.33)					
	Farming	10 (14)		44.30 (4.92)					
	Commercial or service	19 (27)		45.16 (5.22)					
	No job	12 (17)		47.00 (5.34)					
**Experience with internet use**							6.895 (2)		.03
	Less than 1 year	12 (17)		43.00 (5.78)					
	1-3 years	14 (20)		46.93 (4.68)					
	More than 3 years	44 (63)		46.86 (4.45)					
**Experience with mobile phone use**							0.001(1)		.99
	Less than 1 year	0 (0)		0.00 (0.00)					
	1-3 years	6 (9)		45.83 (7.88)					
	More than 3 years	64 (91)		46.25 (4.62)					
**Number of chronic diseases**							0.001 (1)		.98
	1	42 (60)		46.17 (4.51)					
	2 or more	28 (40)		46.29 (5.51)					
**Time postdiagnosis**							1.753 (3)		.62
	Less than 1 year	7 (10)		45.00 (4.00)					
	1-3 years	24 (34)		46.25 (6.04)					
	4-5 years	12 (17)		46.25 (3.62)					
	More than 5 years	27 (29)		46.92 (4.56)					

^a^A paired *t* test was used to obtain this value.

### Behavioral Ability Test

#### Task 1

Of the 70 respondents, 64 (91%) were able to find websites containing health information on the internet, for which, 64% (41/64) chose to enter the URL of the health website directly. Another 36% of respondents (23/64) chose to search for health websites through search engines. Baidu (15/64, 23%) was the most popular search engine, and other search engines were chosen by 13% of respondents (8/64); 9% of respondents (6/70) did not complete this operational task. The primary reason for not completing this task was that they were not in the habit of using computers and mobile phones to search for information, and they just used WeChat for general social interaction.

#### Task 2

A total of 93% of the respondents (65/70) saved articles that they considered valuable. The content of the articles was mainly about disease treatment (73/185, 40%) and health care (54/185, 29%). The rest of the articles did not address health-related issues. However, 35% of the respondents (23/65) had difficulty saving articles because they did not know how to operate an internet browser.

#### Task 3

Similar to operational task 2, 93% of the respondents (65/70) completed this operational task. Among them, 88% (57/65) used search engines (with 30/57, 53% of those who used search engines using the Baidu search engine) to retrieve treatment-related articles for a certain chronic disease; other respondents searched for articles on certain health websites.

Out of a possible score of 75 points on the DISCERN scale, the mean score was 37.4 points (SD 13.8). Out of a possible score of 40 on part 1, the mean score was 23.9 (SD 9.3), and out of a possible score of 35 on part 1, the mean score was 13.6 (SD 6.0); 7 respondents scored below 10 points, 31 respondents scored between 20 and 40 points, and the other respondents scored between 40 and 60 points.

### Semistructured Interviews

A total of 56 respondents completed semistructured interviews (Multimedia Appendix 2).

#### Internet Health Information Retrieval Strategy

##### Usage of Search Engines

The interview results showed that 96% of the interviewees (54/56) preferred to search for health information directly through search engines instead of choosing a special health website. This finding was consistent with the respondents’ performance in operational task 3 and indicated that the awareness rate and selection rate of authoritative health websites were low. Some respondents (20/56, 36%) said the operational task was the first time they had used the internet to find articles about health, indicating that there are still some residents who do not actively use the internet to find health information and knowledge in rural China.

I feel Baidu is more credible in all websites, so need to find some information Baidu has inside.

Participant 7, 45 years old

Because the site involves a wide range of content, the feasibility, desirability, trust found in the viewing are better.

Participant 39, 60 years old

Because the site has the expert review mark, has the doctor basic information and has no advertisement.

Participant 59, 56 years old

##### Choice of Health Website

Although most respondents gave reasons why they chose a particular health information website, including the convenience of searching, personal habits, comprehensive content, and high reliability, 34% of the respondents (19/56) did not know why they chose the website and indicated that their website choice was random. We also assessed how the respondents determined whether a website was the best website to provide health information; 13% of respondents (7/56) believed that a website was the best if it contained a large amount of health information about diseases and treatments, but the respondents were not sure how to determine the authenticity of the articles on the website.

I also did not know which website to choose and had never visited this website before. In this investigation, I chose this website at random, so I could not judge whether this website was good or bad.

Participant 9 years, 44 years old

I have not used Baidu and other search engines, just in the search engine provided health sites, I also randomly selected, for how to evaluate the pros and cons of the site, I do not know.

Participant 9, 44 years old

##### Saving Internet Health Information

A few respondents (4/56, 7%) reported difficulties accessing health websites, which was similar to the findings of operational task 1. However, only 16% of respondents (9/56) thought that they had difficulties saving interesting articles, which was inconsistent with the findings of operational task 2.

It is not difficult to find the site, open a web browser to search for such articles can be easily found. It was not difficult to save articles before, but now I find that some articles need to register and log in when they are saved, or even charge for it, which makes me feel more cumbersome.

Participant 22, 51 years old

#### Evaluation of the Quality of Internet Health Information

##### Layout of the Website

Overall, 75% of the respondents (42/56) thought the layout of the web page was well designed, and 55% of the respondents (31/56) were satisfied with the website in terms of a detailed and clear layout, clear classification, comprehensive search information, provision of answers internet by professional doctors, and a lack of advertising plug-ins and links; however, 32% of the respondents (18/56) were quite dissatisfied with the large number of advertising links interspersed in the web pages.

I feel that the layout design of this kind of health information website is similar. What I am most dissatisfied with is that when I browse the web page, advertisements for doctor consultation often pop up, and they will pop up again and again after I click “close.”

Participant 2, 32 years old

I think the homepage of the website is not bad, and the classification of diseases is obvious. What I am not satisfied with is that the font of the page is relatively small and there are many advertisements, which affect the professionalism.

Participant 61, 47 years old

##### Value of Internet Health Information Content

Of the 56 respondents, 96% (54/56) said they had acquired new knowledge from health information websites, and 6% (36/56) believed that information about disease treatment, rehabilitation, health care and prevention was the most valuable information to them. Additionally, 91% (51/56) said they would visit the website again and recommend it to their relatives and friends.

The most helpful information on this website is the knowledge of disease prevention and improvement. I also learned a lot of knowledge that I didn’t know before from the internet. It is very likely that I will visit this website again, or will I recommend it to my family.

Participant 30, 40 years old

##### Evaluation of the Quality of Internet Health Information

A total of 89% of respondents (50/56) were able to use some method to determine the reliability of the information, while 11% (6/56) said they did not know how to judge the reliability of the various articles on the website.

First of all, the website has the sign of expert review. Secondly, if it is not a regular website, there will be a variety of advertising push, while regular health websites generally have no advertising push.

Participant 59, 56 years old

#### Comments and Suggestions

**Figure 1:**
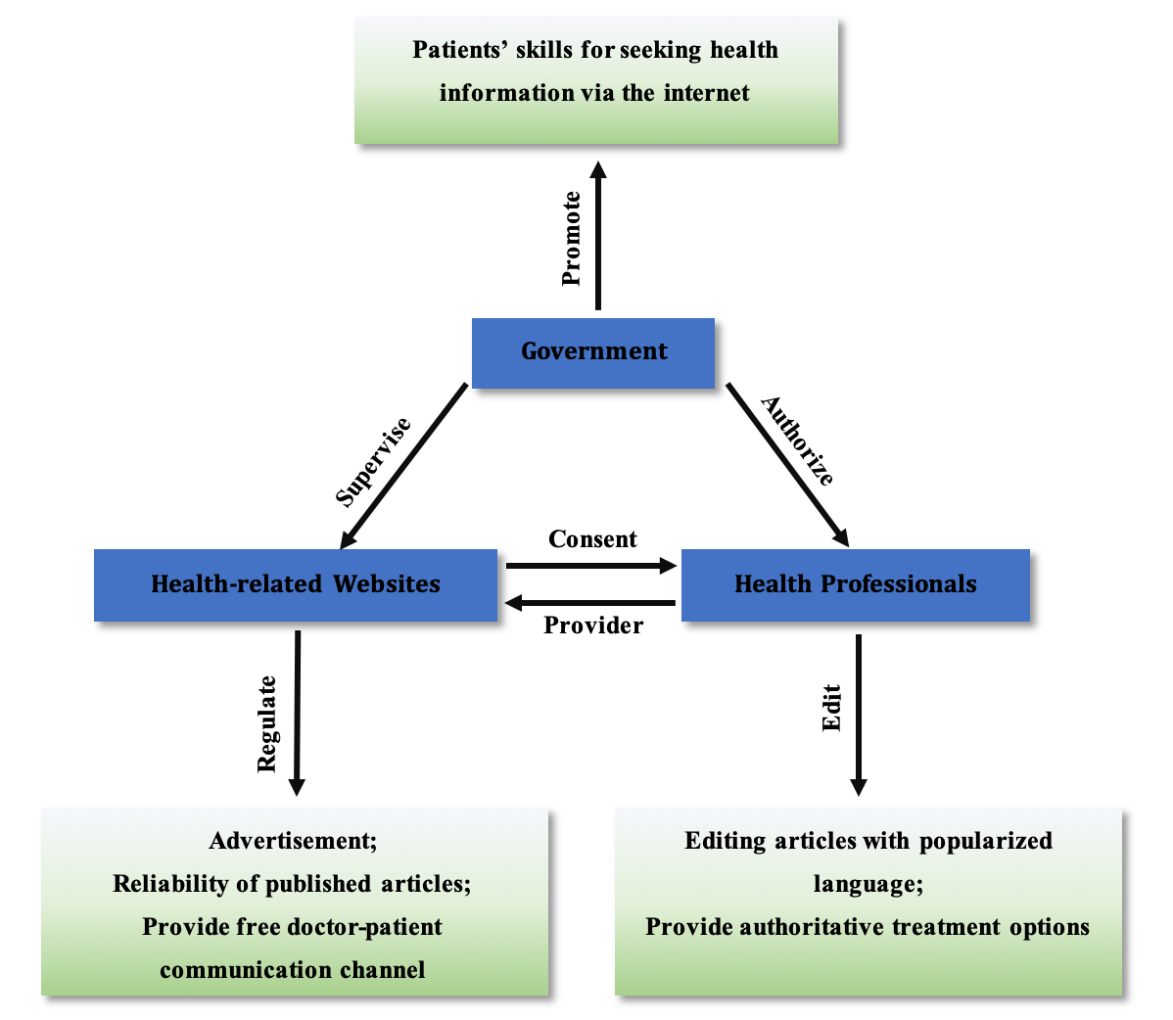
Interviewee suggestions.

At the end of the interview, we conducted an analysis of the interview recordings and summarized the interviewees' opinions and suggestions on obtaining internet health information (Figure 1).

## Discussion

### Principal Results

In this study, qualitative and quantitative research methods were used to investigate the status quo of access to internet health information among 70 rural community patients with chronic diseases to comprehensively assess their health information literacy levels and ability to access internet health information and to summarize the obstacles they faced in accessing internet health information.

The self-rated health information literacy score of all the interviewees was above 30 points, with more than 90% of respondents (63/70) scoring above 40, indicating that most respondents had a moderate health information literacy level. The health information literacy levels of the respondents over 60 years old were lower than those of younger respondents, and there were significant differences in the health information literacy scores of the respondents of different sexes, education levels and internet use experiences, which is similar to the findings of previous studies [18,20,21]. Health information literacy is an important aspect of individual cultural literacy, and there is an interactive relationship between health information attitude and health information skills [27]. Therefore, an important aspect of future health education and health promotion is enhancing the awareness of patients with chronic disease on the value of health information and cultivating their practical ability to obtain internet health information.

The results of operational task 1 and operational task 2 indicated that most respondents had a low ability to access health information. The respondents randomly chose health websites and could not judge whether the website was reliable based on the number of visits to the website and the authority of the articles. Most articles selected by the respondents came from websites with encyclopedic knowledge or answers from people based on their own experiences rather than authoritative health information websites. The respondents’ abilities to download or save interesting articles were poor. One important reason for this finding is that people's use of the internet is mostly for entertainment and leisure (such as using Tiktok and WeChat [28]). This only requires people to know the basics of Android or iOS (such as opening an app, returning to the home page, and selecting and typing text). People use their mobile phones as a tool to query internet health information only when health problems occur [29], and proficiency of this tool requires the support of certain operational abilities (such as using the browser, opening a web page, downloading files, saving files, setting up software).

In terms of the quality of the internet health information obtained, most health-related articles saved by the respondents did not specify the author, publisher, publication date, or references. The articles obtained via search engines were primarily written based on people’s experiences, and most treatment protocols involved were based on the subjective opinions of individuals and failed to describe the effects and risks of the treatment methods. Several articles published on websites not related to medical health were even chosen as describing the best treatment options. Although a large number of internet evaluation tools have been developed, these tools are mostly designed from the perspective of expert evaluation, and there are few public-facing evaluation systems [30]. Therefore, it is necessary to develop public-facing internet health information quality evaluation tools as soon as possible. Additionally, the results of the DISCERN evaluation indicated that most respondents lacked the skills to access high-quality health-related information via the internet. Combining the results of the health literacy self-assessment with those of the DISCERN evaluation, we found that most respondents overrated their ability to seek high-quality health information internet.

In further conversations with 56 of the respondents, we found that most had directly retrieved health-related information through search engines (such as Baidu), while the proportion of health-related information obtained from professional health sites and medical sites was relatively low. Most interviewees believed that they could retrieve professional health information from the websites. The popularity of the websites, the quality of the articles, the illness of the interviewees and the advice of their doctors were the main reasons for their choice of internet health information. While 16% of interviewees (9/56) admitted that they had difficulties saving and downloading health information, this proportion reached 35% (23/65) in operational task 2, which contradicted the respondents’ self-assessments. The respondents' evaluation of internet health information quality relied more on their own judgements than on other factors because they did not compare health information from multiple channels and seldom consulted professionals. Most respondents rated health-related websites based on the reasonableness of the page layout and whether there were spam ads. They were more concerned with the richness and comprehensibility of the website content than the authenticity and reliability of the content. This finding indicates that there is a need to strengthen education on the awareness for internet health information evaluation among patients with chronic diseases. Simultaneously, as providers of internet health information, health websites should also strengthen quality control of their health information and improve the publicity of their website so that consumers can more easily find the high-quality internet health information that they need.

### Limitations

This study had some limitations. First, this study investigated only 70 patients with chronic disease in 10 communities of a small town near Bengbu City. The sample size was relatively small, and the scope of the research was limited, which limits the generalizability of the results to some extent. Second, this study designed only 3 tasks to evaluate the residents' behavioral ability to obtain internet health information, which limits the ability to fully reflect the actual level of rural residents' behavioral abilities to obtain internet health information. Finally, some bias was inevitable due to the influence of differences in social and cultural backgrounds between interviewers and interviewees who understand the questions differently during the semistructured interview process. Therefore, the results of this study should be regarded as preliminary and interpreted with caution. Despite these limitations, the findings of this study are valuable for understanding the internet health information–seeking behavior of patients with chronic diseases in rural China and their attitudes about internet health information.

### Practical Implications

Governments and relevant departments should strengthen education for the general public to help them increase their ability to access internet health information and access free and high-quality internet health information resources. Governments and relevant departments should also increase supervision on the release and dissemination of internet health information with relevant laws and regulations to improve the internet health information environment.

### Conclusion

We found that the level of internet information access among patients with chronic disease living in rural China was moderate. The vast majority of the respondents recognized the importance of accessing health information but were not very proactive in accessing health information via the internet. Furthermore, there was a gap between their actual ability to access high-quality internet health information and their self-rated health information literacy. Most respondents experienced difficulties seeking internet health information, and they lacked the skills to screen for high-quality internet information. Although most interviewees listed certain methods for judging the quality of internet health information, the behavioral ability test showed that they did not follow the expected methods to obtain high-quality internet health information but rather only made subjective judgements and showed a certain degree of randomness in their selection processes.

## Appendix

Multimedia Appendix 1 Investigation materials for patients with chronic diseases.

Multimedia Appendix 2 Analysis of the semistructured interview results of the 56 interviewees.

## Abbreviations

RMB: Renminbi
