# Supplementary material for: The Gap Between Self-Rated Health Information Literacy and Internet Health Information-Seeking Ability for Patients With Chronic Diseases in Rural Communities: Cross-sectional Study
Source: J Med Internet Res. 2022 Jan 31;24(1):e26308. doi: 10.2196/26308 (PMC8845012; doi:10.2196/26308)
Supplement: Multimedia Appendix 1 [file jmir_v24i1e26308_app1.docx]

Annex I: Investigation materials for patients with chronic diseases

Research profile

Purpose of study: The purpose of our study was to investigate how people with chronic diseases access and evaluate health information on the Internet. Because you meet the inclusion criteria of our research sample, we invite you to participate in our study.

Research content: This research includes three contents. First, you are asked to complete a health information literacy self-assessment questionnaire (containing 14 questions).Then, you are invited to complete three tasks of chronic health information retrieval through the Internet. We will record your online behavior through screen recording software and analyze your search process. When you have finished browsing the Internet, we will conduct an interview with you (including 9 questions). Finally, please give an overall evaluation on the access to chronic disease health information through the Internet.

Informed Consent: Before starting this study, please take a few minutes to read the informed consent form below and sign it in person. Please sign your name and agree to participate in our study. We can provide you with a copy of the informed consent if you require it.

Informed consent

This survey is the first part of the research work of the key research project of Humanities and Social Sciences of Education Department of Anhui Province, health Information Literacy Education Path research for Chronic disease Patients facing General practitioners. The aim is to understand the ability of people with chronic diseases to access health information through the Internet and the problems they may encounter.

During the survey, the whole process of the interviewees obtaining health-related information through the Internet will be recorded on the screen, and the interview process will be recorded, so that the survey results can be clearly and accurately analyzed after the survey is completed.

The research group promises to keep strictly confidential all the audio and video recordings and questionnaire data involved in the research process.

I have fully understood the relevant matters of this investigation and agree to participate in this investigation.

Signature:

Date:

1. Self-evaluation of health information literacy

The following 14 questions are for understanding your health information literacy status. There is no right or wrong answer. Please choose the appropriate answer according to your situation.

| （1）Do you know where you can get health-related knowledge? | | | | | | |
| --- | --- | --- | --- | --- | --- | --- |
| Completely know | Know some | Don't know much about it | | | | Don't know |
| （2）Do you find it easy to access health information through the Internet? | | | | | | |
| Very easy to | Relatively easy to | It is a little hard | | | | It is very difficult |
| （3）Do you find it easy to get health information through print media (magazines or books)? | | | | | | |
| Very easy to | Relatively easy to | It is a little hard | | | | It is very difficult |
| (4) Do you think it is easy to get health information through TV? | | | | | | |
| Very easy to | Relatively easy to | It is a little hard | | | | It is very difficult |
| (5) Do you feel that terms and sentences related to health information are often difficult to understand? | | | | | | |
| It's easy to understand | Basically understand | | Don't understand | Basically can't understand | | |
| (6) Do you find it easy to evaluate the reliability of health information in print media (magazines or books)? | | | | | | |
| Very easy to | Relatively easy to | It is a little hard | | | | It is very difficult |
| (7) Do you find it easy to evaluate the reliability of health information on the Internet? | | | | | | |
| Very easy to | Relatively easy to | It is a little hard | | | | It is very difficult |
| (8) Do you find it easy to evaluate the reliability of health information on television? | | | | | | |
| Very easy to | Relatively easy to | It is a little hard | | | | It is very difficult |
| (9) When discussing health-related issues with others, do you often feel like you don't know whose word to believe? | | | | | | |
| Completely know | Basic know | | I'm not sure | | Have no idea | |
| (10) Do you agree that health-related knowledge and information is important to keep you healthy? | | | | | | |
| Well accepted | Basic agree | | Don't recognized | | Do not recognise | |
| (11) Would you like to have access to health-related knowledge and information from multiple sources? | | | | | | |
| Very much hope | Hope | | It doesn't matter | | Don't want to | |
| (12) If your body encounters a health problem, can you express the health information you wish to obtain? | | | | | | |
| Can express | Can express, but a little difficult | | Barely able to express | | Have no idea how to express it | |
| (13) Will you apply your health-related knowledge or information to yourself and those around you? | | | | | | |
| Very glad to | It doesn't matter | | Basic won't | | Not completely | |
| (14) Are you willing to share your health knowledge or information with others? | | | | | | |
| Very glad to | It doesn't matter | | Reluctant to | | Don't want to | |

2. Online health information retrieval behavior of chronic diseases

Please complete the following three health information retrieval tasks via the Internet:

(1) Use the Internet to find a site that you think contains the best health information;

(2) Select 3 articles from this site that are related to your health and are of the greatest interest to you, and save them to your local disk;

(3) Choose a chronic disease according to your health condition, and use the Internet to find the treatment plan you think is the best;

3. Interview

Now that you have completed the online chronic health information retrieval task, I would like to ask you some questions about this process and answer my questions as best you can. There is no right or wrong answer to the question. We just want to know what you think. Please speak loudly and clearly so that when we listen, we can clearly play back your answers and analyze the information.

3.1. Retrieval process and strategy

A. For the website (or information publishing platform) where you find the best chronic disease health knowledge, how do you determine if it is the best website for chronic disease knowledge?

B_1_. If you use a web search engine, answer the following question: Have you used this search engine before? Why did you choose this site over the other sites offered by the search engines? Have you visited this site before?

B_2_. If you are not using a search engine, please answer the following question: How did you find out about this website? Have you visited this site before?

C. In general, do you find it easy or difficult to find this website?

D. Do you find it difficult to download or save articles on chronic diseases of interest? What steps did you find difficult?

3.2. Site assessment

A. what is your first impression of this website (or mobile messaging platform)? What do you think of the layout of the homepage content on the website (or mobile messaging platform)?

B. What aspects (features) of the design of this website (or information publishing platform) do you think would make you approve of it? What aspects (features) of the design of the website (or information distribution platform) are you most dissatisfied with?

C. What is the most helpful information on this website (information dissemination platform) in terms of understanding chronic diseases? Does this site provide information about chronic health conditions that you were previously unaware of?

D. With regard to the treatment programme for chronic diseases, what are the factors by which you judge the reliability of the programme?

E. Would you visit this website (or information distribution platform) again? Would you refer a family member or friend to this website (or information distribution platform) if they wanted to learn about chronic diseases? Why?

4. Do you have any other questions about accessing chronic health information through the Internet?

5. Basic Information

Age:

Gender:

| □ Male | □ Female |
| --- | --- |

Education:

| □Primary school and below |  |
| --- | --- |
| □middle school/technical school/technical secondary school | |
| □ University | □ postgraduate and above |

Nature of work:

| □ Employees of enterprises and public institutions (including separation and retirement) | |
| --- | --- |
| □ farming | □ self-employed |
| □ no job |  |

Duration of Internet use:

| □ Never used | □ less than 1 year |
| --- | --- |
| □ 1-3 years | □ more than 3 years |

Cell phone usage time

| □ Never used | □ 1 year to 3 years |
| --- | --- |
| □ More than 3 years |  |

Situation of chronic diseases

| □ Suffering from a chronic disease | □ Suffering from two or more chronic diseases |
| --- | --- |

The time of first diagnosis of a chronic disease

| □Within 1 year | □1 year to 3 years |
| --- | --- |
| □3 years to 5 years | □More than 5 years |

Has been to receive gifts, please signature:

tel:

The investigation is now over. Wish you good health!
