# Supplementary material for: The Gap Between Self-Rated Health Information Literacy and Internet Health Information-Seeking Ability for Patients With Chronic Diseases in Rural Communities: Cross-sectional Study
Source: J Med Internet Res. 2022 Jan 31;24(1):e26308. doi: 10.2196/26308 (PMC8845012; doi:10.2196/26308)
Supplement: Multimedia Appendix 2 [file jmir_v24i1e26308_app2.docx]

Annex II: Analysis of the semi-structured interview results of the 56 interviewees

|  | **Problem** | **Result** | **Analysis** |
| --- | --- | --- | --- |
| Retrieval process  and strategy | 1. For the website (or information publishing platform) where you found the best chronic disease health knowledge, how did you judge that this website was the best one for providing knowledge of chronic diseases? | Twenty respondents (35.7%) made judgements based on comparison with other websites, their state of illness, and a general practitioner’s advice; 12 (21.4%) made judgements based on the quality of the website content; 7 (12.5%) made judgements based on the popularity of the website; 6 (10.7%) made judgements based on the source of the article; 4 (7.1%) said that the information on the Internet was only for reference; 5 (8.9%) did not know how to judge the website; and 2 (3.6%) did not report their views. | Judgement criteria: high website popularity, good netizen evaluations, official authoritative and reliable website sources, rich website content, few advertisements, high-quality articles, consistent with the respondent’s condition, etc. |
|  | 2.1 If you used a web search engine, please answer the following questions: Have you used this search engine before? Why did you choose this website instead of others? Have you visited this site before?  2.2. If you did not use a search engine, please answer the following question: How did you know about this website? Have you visited this website before? | Thirty-two respondents (57.1%) used the Baidu search engine, 12 (21.4%) used the 360 search engine, 10 (17.9%) used the Sogou search engine, and 2 (3.6%) did not use a search engine and only used a WeChat public account. Of those who used search engines, 43 (76.8%) were regular users of search engines.  Twelve respondents (21.4%) followed a personal habit; 9 (16.1%) affirmed the content of the website; 8 (14.3%) thought it was more convenient to find information on the website; 8 (14.3%) were positive about the credibility of the site; 4 (7.1%) randomly selected the site; and 15 (26.8%) were unclear. Of those who did not use a search engine, 36 (64.3%) had visited the site before, and 20 (35.7%) have not visited the site before. | The main reason why respondents chose search engines to find health information was that search engines could quickly access multiple pieces of relevant information without requiring specialized search skills.  Selection criteria: Ease of access of the website, authoritative official website, comprehensive details on the website, and good correspondence with the respondent’s condition. |
|  | 3. Generally, did you think it was easy or difficult to find this website? | Four respondents (7.1%) had some difficulties finding the website | The primary difficulty was that they did not know how to use a browser and save article links, and some articles were not free to download. |
|  | 4. Did you have difficulties downloading or saving the chronic disease articles that you were interested in? Which steps did you think were difficult? | Nine respondents (16.1%) had difficulties downloading or saving articles. |  |
| Evaluation of the quality of online health information | 1. What was your first impression of this website (or mobile messaging platform)? What do you think of the layout of the homepage content on the website (or mobile messaging platform)? | First impression: 52 respondents (92.9%) thought the website was not bad; 2 (3.6%) thought the information on the website had low credibility and false information, and 2 (3.6%) did not report a definite attitude.  Forty-two respondents (75%) thought that the website layout was reasonable, 4 (7.1%) thought that spam advertisements interfered with reading, 3 (5.4%) did not pay attention to this aspect, and 7 (12.5%) did not report a definite attitude. | First impressions: the layout of the health information site was well organized; 7.1% of the respondents felt that although the layout was reasonable, there are still have spam advertisements. |
|  | 2. What aspects (features) of the design of this website (or information publishing platform) did you think would make you approve of it? What aspects (features) were you most dissatisfied with in the design of the website (or information publishing platform)? | Twenty-six respondents (46.4%) were satisfied with the high search efficiency and provision of comprehensive and understandable health information, 5 (9%) were satisfied with the online question and answer section, and 25 (44.6%) did not give a clear answer.  18 (32.1%) were dissatisfied with the spam advertisements that popped up on the website; 38 (67.9%) thought they were very satisfied. | Factors related to satisfaction: detailed and clear interface, explicit and appropriate category page, coverage of a wide range of health information, answers written by a professional physician online, no spam plug-ins or links, etc.  Factors related to dissatisfaction: many pop-up advertising links and consultations that affected the reading of articles and false propaganda. |
|  | 3. What was the most helpful information on this website (information dissemination platform) in terms of understanding chronic diseases? Did this website provide health information about chronic diseases that you did not know before? | Twenty-four respondents (42.9%) found information on the therapeutic schedule for chronic diseases to be most helpful, 11 (19.6%) found information on rehabilitation care to be most helpful, 5 (8.9%) found information on disease prevention to be most helpful, 16 (28.6%) did not give a clear answer, 54 (96.4%) indicated that they had learned new knowledge and 2 (3.6%) did not report a definite attitude. | The respondents thought health websites should make every effort to help them by providing a treatment plan of disease, relevant knowledge on disease prevention and rehabilitation care, and details on how to obtain new information from the website. |
|  | 4. What factors did you use to estimate the reliability of the information on the therapeutic schedule for chronic diseases? | Sixteen respondents (28.6%) compared the information with their symptoms, 10 (17.9%) consulted a general practitioner, 9 (16.1%) made judgements based on their feelings, 6 (10.7%) judged whether the content of the website was objective and professional, 5 (8.9%) made judgements based on their practices, 4 (7.1%) made judgements by comparing with the information with information from other websites, and 6 (10.7%) were not sure how to judge the reliability. | The respondents' main judgement methods: comparing the information with their state of illness, consulting a general practitioner, trying the recommendations oneself, examining the corresponding therapeutic schedule for chronic diseases on the website, and seeking expert advice. |
|  | 5. Would you visit this website (or information distribution platform) again? Would you recommend this website (or information distribution platform) to family members or friends if they wanted to learn about chronic diseases? Why? | Fifty-one respondents (91%) said they would visit the website again, 2 (3.6%) they would not visit again, and 3 (5.4%) did not report a definite attitude; 51 (91%) said they would recommend it, 2 (3.6%) would not recommend it, and 3 (5.4%) did not report a definite attitude | The respondents who said they would visit the website again found the website easy to use, thought it provided professional information, and were willing to recommend it to others.  The respondents who said they would not visit the website again said that the information they learned from the Internet was shallow and untrustworthy, and therefore, they would not recommend it to others. |
